# Comparative speed of kill of oral treatments with Simparica^TM^(sarolaner) and Bravecto®(fluralaner) against induced infestations of *Rhipicephalus sanguineus* on dogs

**DOI:** 10.1186/s13071-016-1376-x

**Published:** 2016-02-24

**Authors:** Csilla Becskei, Thomas Geurden, Julian Liebenberg, Otto Cuppens, Sean P. Mahabir, Robert H. Six

**Affiliations:** Zoetis, Veterinary Medicine Research and Development, Mercuriusstraat 20, B-1930 Zaventem, Belgium; ClinVet International (pty) Ltd, Uitsigweg, Bainsvlei, 9338 Bloemfontein Republic of South Africa; Zoetis, Veterinary Medicine Research and Development, 333 Portage St., Kalamazoo, MI 49007 USA

**Keywords:** *Rhipicephalus sanguineus*, Sarolaner, Simparica^TM^, Fluralaner, Speed of kill, Tick, Dog, Oral, Isoxazoline, Bravecto®

## Abstract

**Background:**

*Rhipicephalus sanguineus* is the most widely distributed tick species infesting dogs worldwide, which may cause discomfort to the host and transmit diseases. Acaricides with a rapid and sustained speed of kill are thus important to prevent infestation and to reduce the risk of disease transmission. In this study, the speed of kill of a monthly administered Simparica^TM^(sarolaner) treatment against induced infestations with *R. sanguineus* on dogs was evaluated and compared with a single dose of Bravecto®(fluralaner) for 95 days after the initial treatment.

**Methods:**

Twenty four dogs were randomly allocated to treatment and were treated with either placebo or sarolaner (at 2 to 4 mg/kg) on Days 0, 30 and 60 or with fluralaner (at 25 to 56 mg/kg) once on Day 0. Tick counts were performed *in situ* 8 and 12 h and with removal of the ticks 24 h after treatment and subsequent re-infestations on Days 14, 28, 44, 56, 74, 90 and 95. Acaricidal efficacy was determined at each time point relative to the placebo group.

**Results:**

Both products significantly reduced live ticks within 8 h after treatment against an existing infestation with *R. sanguineus*, and killed all ticks on all dogs within 24 h. After re-infestation, sarolaner provided ≥98.5 % reduction within 24 h on all days except Days 74 and 95 (*P <* 0.0001), compared to fluralaner which provided ≥95.5 % reduction until Day 44. Geometric mean live tick counts for sarolaner were significantly lower (*P ≤* 0.0415) at 24 h than those for fluralaner on all days, except on Days 0, 14 and 28 (*P ≥* 0.0678). There were no treatment-related adverse reactions observed during the study.

**Conclusions:**

When dosed at monthly intervals for 3 consecutive months, Simparica^TM^ has a faster and more consistent speed of kill against *R. sanguineus* than a single oral dose of Bravecto® for which efficacy decreased after Day 44.

## Background

*Rhipicephalus sanguineus,* also known as the kennel tick or brown dog tick*,* has a worldwide distribution in areas with a relatively warm climate and mild winters [1, 2]. The introduction of *R. sanguineus* in regions with less favorable conditions has been described, mainly through dogs returning from travel in endemic areas [3–7]. As the tick is known to complete the life cycle in-house and as climate and environmental conditions become more suitable [7, 8], expansion to new distribution areas is anticipated. *Rhipicephalus sanguineus* is prevalent both in rural and urban areas [1, 9], and is a known vector for several vector-borne pathogens*,* including *Ehrlichia* spp*., Babesia canis, Hepatozoon canis* and several *Rickettsia* spp. [1, 10]. The awareness of and exposure to tick-borne diseases increases and has driven the emphasis on tick control and prevention in recent years. Simparica^TM^(sarolaner) is an oral isoxazoline parasiticide that provides excellent treatment and prevention of ticks, including *R. sanguineus,* for at least 5 weeks after a single oral dose [11]. Although the systemically active compounds require the tick to bite to be effective, the isoxazolines are known to act rapidly. A single dose of sarolaner provides adequate efficacy against *Ixodes ricinus, I. scapularis, Dermacentor reticulatus* and *Amblyomma maculatum* within 24 h after treatment or re-infestation for at least 4 weeks [12, 13]. The present study aimed to evaluate and compare the speed of kill of a monthly oral dose of sarolaner with a single oral dose of Bravecto® (fluralaner) against an existing infestation and against re-infestations with *R. sanguineus* for a period of 95 days.

## Methods

### Ethical approval

Study procedures were in accordance with the World Association for the Advancement of Veterinary Parasitology (WAAVP) guidelines for evaluating the efficacy of parasiticides for the treatment, prevention and control of flea and tick infestation on dogs and cats [14]. The protocol was reviewed and approved by the Zoetis Ethical Review Board and local Animal Welfare Committee. Masking of the study was assured through the separation of study functions. All personnel conducting observations or animal care or performing infestations and counts were masked to treatment allocation.

### Animals

Twenty-four (12 male and 12 female) purpose-bred Beagle and mixed breed dogs from 11 months to 9 years of age and weighing from 9.4 to 22.8 kg were used in the study. Each dog had undergone an adequate wash-out period to ensure that no residual ectoparasiticide efficacy remained from any previous treatment. Dogs were individually housed and they were acclimatized to these conditions for 8 days prior to treatment. Dogs were fed an appropriate maintenance ration of a commercial canine feed for the duration of the study. Water was available *ad libitum*. All dogs were given a physical examination to ensure that they were in good health at enrollment and were suitable for inclusion in the study. General health observations were performed twice daily throughout the study.

### Design

The study followed a randomized complete block design. Dogs were ranked according to decreasing pre-treatment tick counts (48 h after infestation on Day-7) into blocks of three animals, and within each block a dog was randomly allocated to one of three treatment groups. There were eight dogs per treatment group.

### Treatment

On Days 0, 30 and 60, two groups of dogs each received either a placebo tablet or an appropriate Simparica^TM^ chewable tablet to provide label dose (sarolaner at 2 to 4 mg/kg). The third group of dogs received a Bravecto® tablet (per label providing fluralaner at 25 to 56 mg/kg) on Day 0 and placebo tablets on Days 30 and 60. In order to comply with the Bravecto® label requirement, all dogs were fed within 20 min of the treatment administration. The tablet(s) were administered by hand pilling to ensure accurate and complete dosing. Each dog was observed for several minutes after dosing for evidence that the dose was swallowed, and for potential adverse events associated with treatment administration. Dogs were observed approximately two hours after dosing for evidence of emesis.

### Tick infestation and assessment

The *R. sanguineus* strain used for infestation was originally isolated from the field in France in 2007. The colony was genetically enriched by the addition of wild-caught ticks from France in 2012. For treatment allocation dogs were infested with ticks on Day -7 and ticks counts were conducted 48 h later. Further tick infestations were performed on Days -2, 14, 28, 42, 56, 74, 90 and 95. Prior to each infestation, the dog was sedated to enhance tick attachment, and 50 (±5) viable unfed adult *R. sanguineus* (1:1 male:female) were directly applied to each animal. Tick counts were conducted at 8, 12 and 24 (±0.5) hours after treatment and each subsequent weekly re-infestation. Ticks were counted *in situ* (thumb counts) at the 8 and 12 h time points by systematic examination of the entire body surface to ensure that any area was examined only once. At the 24 h counts, dogs were examined and then thoroughly combed to count and remove ticks. Each dog was examined for at least 10 min. If ticks were encountered in the last minute, combing was continued in one minute increments until no ticks were encountered.

### Statistical analysis

The individual dog was the experimental unit. Data for post-treatment live (free plus attached) tick counts were summarized with arithmetic (AM) and geometric (GM) means by treatment group and timepoint. Tick counts were transformed by the log_e_(count + 1) transformation prior to analysis in order to stabilize the variance and normalize the data. Using the PROC MIXED procedure (SAS 9.2, Cary NC), transformed counts were analyzed using a mixed linear model for repeated measures. The fixed effects were treatment, time-point and the treatment by time-point interaction. Random effects included room, block within room, block by treatment interaction within room, and error. Testing was two-sided at the significance level α = 0.05. The assessment of acaricidal efficacy was based on the percent reduction in the arithmetic and geometric mean live tick counts relative to placebo [15], and was calculated using Abbott’s formula:

$$ \%\ \mathrm{reduction}=100 \times \frac{\mathrm{mean}\ \mathrm{count}\ \left(\mathrm{placebo}\right)\hbox{--}\ \mathrm{mean}\ \mathrm{count}\ \left(\mathrm{treated}\right)}{\mathrm{mean}\ \mathrm{count}\ \left(\mathrm{placebo}\right)} $$

## Results

**Fig. 1:**
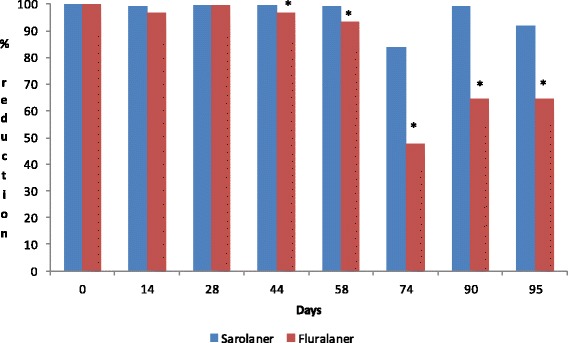
Percent efficacy based on geometric mean *Rhipicephalus sanguineus* counts relative to placebo at 24 h after treatment and weekly re-infestation for dogs receiving a monthly oral dose of sarolaner on Days 0, 30 and 60 or a single oral dose of fluralaner on Day 0

The results of the tick counts at each time-point are provided in Tables 1, 2 and 3, and in Fig. 1. Placebo-treated dogs maintained adequate tick infestations throughout the study. Both products significantly reduced tick counts within 8 h after the initial treatment (*P <* 0.0001) and achieved >90 % efficacy by 12 h against the existing infestation. At 8 and 12 h after subsequent weekly re-infestations both products had a variable speed of kill and efficacy tended to decline towards the end of the treatment period with tick counts for both groups not being significantly different from placebo on Days 58, 74 and 95 (Tables 1 and 2).

**Table 1:** Efficacy of sarolaner after three monthly treatments on Days 0, 30 and 60 and after a single oral dose of fluralaner on Day 0 against *Rhipicephalus sanguineus* on dogs 8 h after treatment and re-infestations (AM: arithmetic mean live tick counts; GM: geometric mean live counts)

Treatment		Day of infestation							
		0	14	28	44	58	74	90	95
Placebo	Range	15 to 34	15 to 38	20 to 39	15 to 37	12 to 30	19 to 36	19 to 28	21 to 41
	AM	20.5	23.8	32.3	28.0	24.3	28.8	23.9	31.8
	GM^d^	19.8^a^	22.8^a^	31.6^a^	27.3^a^	23.3^a^	28.2^a^	23.7^a^	31.3^a^
Sarolaner	Range	0 to 33	11 to 25	9 to 19	9 to 23	11 to 29	17 to 49	8 to 20	21 to 36
	AM	6.3	14.5	14.1	14.0	20.0	31.6	12.1	26.8
	AM Efficacy (%)	69.5	38.9	56.2	50.0	17.5	0.0	49.2	15.7
	GM^d^	2.7^b^	14.0^b^	13.8^b^	13.5^b^	19.0^a^	30.4^a^	11.7^b^	26.3^a^
	GM Efficacy (%)	86.6	38.5	56.3	50.6	18.5	0.0	50.8	16.0
	*P*-value vs. placebo	<0.0001	0.0126	<0.0001	0.0003	0.2857	0.6949	0.0004	0.3561
Fluralaner	Range	0 to 10	9 to 17	6 to 16	9 to 24	14 to 28	21 to 34	5 to 23	21 to 39
	AM	2.3	13.3	10.0	16.5	23.0	26.0	13.8	25.4
	AM Efficacy (%)	89.0	44.2	69.0	41.1	5.2	9.6	42.4	20.1
	GM^d^	1.3^c^	13.0^b^	9.5^b^	15.7^b^	22.5^a^	25.6^a^	12.8^b^	24.9^a^
	GM Efficacy (%)	93.6	43.0	69.9	42.4	3.4	9.3	46.1	20.3
	*P*-value vs. placebo	<0.0001	0.0050	<0.0001	0.0055	0.8580	0.6132	0.0021	0.2415
	*P*-value vs. sarolaner	0.0433	0.7638	0.1461	0.5413	0.4936	0.4822	0.7219	0.8301

^d^Geometric means within a counting day with the same superscript are not significantly different (*P* > 0.05)

**Table 2:** Efficacy of sarolaner after three monthly treatments on Days 0, 30 and 60 and after a single oral dose of fluralaner on Day 0 against *Rhipicephalus sanguineus* on dogs 12 h after treatment and re-infestations (AM: arithmetic mean live tick counts; GM: geometric mean live counts)

Treatment		Day of infestation							
		0	14	28	44	58	74	90	95
Placebo	Range	11 to 35	17 to 37	20 to 39	24 to 37	13 to 26	12 to 34	20 to 26	20 to 38
	AM	20.8	24.1	31.6	28.3	21.4	26.8	22.3	29.1
	GM^d^	19.7^a^	23.5^a^	31.1^a^	28.0^a^	20.8^a^	25.8^a^	22.2^a^	28.7^a^
Sarolaner	Range	0 to 11	2 to 12	7 to 14	5 to 14	9 to 23	12 to 38	5 to 22	20 to 33
	AM	1.6	8.0	10.0	10.0	17.0	26.9	12.1	25.5
	AM Efficacy (%)	92.2	66.8	68.4	64.6	20.5	0.0	45.5	12.4
	GM^d^	0.6^b^	7.3^b^	9.7^b^	9.6^b^	16.3^a^	25.7^a^	11.1^b^	25.1^a^
	GM Efficacy (%)	96.8	69.0	68.7	65.7	21.6	0.3	49.8	12.4
	*P*-value vs. placebo	<0.0001	<0.0001	<0.0001	<0.0001	0.2065	0.9863	0.0005	0.4861
Fluralaner	Range	0 to 2	2 to 11	3 to 10	5 to 19	9 to 24	19 to 32	5 to 17	21 to 29
	AM	0.4	5.9	5.8	11.0	16.4	24.4	12.8	24.9
	AM Efficacy (%)	98.2	75.6	81.8	61.1	23.4	8.9	42.7	14.6
	GM^d^	0.3^b^	5.3^b^	5.3^c^	9.9^b^	15.4^a^	23.9^a^	12.1^b^	24.7^a^
	GM Efficacy (%)	98.7	77.5	83.1	64.5	26.1	7.3	45.4	13.7
	*P*-value vs. placebo	<0.0001	<0.0001	<0.0001	<0.0001	0.1267	0.6959	0.0027	0.4485
	*P*-value vs. sarolaner	0.2720	0.2450	0.0238	0.8946	0.8149	0.7673	0.7431	0.9509

^d^Geometric means within a counting day with the same superscript are not significantly different (*P* > 0.05)

**Table 3:** Efficacy of sarolaner after three monthly treatments on Days 0, 30 and 60 and after a single oral dose of fluralaner on Day 0 against *Rhipicephalus sanguineus* on dogs 24 h after treatment and re-infestations (AM: arithmetic mean live tick counts; GM: geometric mean live counts)

Treatment		Day of infestation							
		0	14	28	44	58	74	90	95
Placebo	Range	14 to 36	21 to 39	21 to 39	21 to 37	14 to 29	18 to 36	22 to 31	25 to 37
	AM	23.5	26.3	28.8	28.0	25.0	29.0	25.8	30.5
	GM^d^	22.8^a^	25.8^a^	28.2^a^	27.6^a^	24.4^a^	28.3^a^	25.6^a^	30.2^a^
Sarolaner	Range	0 to 0	0 to 1	0 to 1	0 to 2	0 to 1	1 to 11	0 to 2	0 to 11
	AM	0.0	0.3	0.1	0.3	0.3	5.3	0.4	3.8
	AM Efficacy (%)	100	99.0	99.6	99.1	99.0	81.9	98.5	87.7
	GM^d^	0.0^b^	0.2^b^	0.1^b^	0.1^c^	0.2^c^	4.6^c^	0.3^c^	2.5^c^
	GM Efficacy (%)	100	99.3	99.7	99.5	99.2	83.8	99.0	91.9
	*P*-value vs. placebo	<0.0001	<0.0001	<0.0001	<0.0001	<0.0001	<0.0001	<0.0001	<0.0001
Fluralaner	Range	0 to 0	0 to 3	0 to 1	0 to 5	0 to 8	4 to 27	4 to 20	2 to 32
	AM	0.0	1.1	0.1	1.3	3.1	16.8	10.8	14.8
	AM Efficacy (%)	100	95.7	99.6	95.5	87.5	42.2	58.3	51.6
	GM^d^	0.0^b^	0.8^b^	0.1^b^	0.9^b^	1.6^b^	14.8^b^	9.1^b^	10.8^b^
	GM Efficacy (%)	100	96.8	99.7	96.9	93.5	47.9	64.6	64.3
	*P*-value vs. placebo	<0.0001	<0.0001	<0.0001	<0.0001	<0.0001	0.0011	<0.0001	<0.0001
	*P*-value vs. sarolaner	1.0000	0.0678	1.0000	0.0415	0.0012	<0.0001	<0.0001	<0.0001

^d^Geometric means within a counting day with the same superscript are not significantly different (*P* > 0.05)

Twenty-four hours after treatment, no live ticks were found on any sarolaner or fluralaner-treated dog (100 % efficacy; *P <* 0.0001 vs placebo; Table 3). Twenty-four hours after subsequent re-infestations, the reduction in AM (GM) tick counts for sarolaner was above 98.5 % (99.0 %) for 4 weeks after each monthly treatment, except on Day 74 when efficacy was 81.9 % (83.8 %). GM live tick counts for sarolaner were significantly lower (*P <* 0.0001) than placebo on all days. The reduction in AM (GM) tick counts for fluralaner was ≥95.5 % (96.9 %) until Day 44, and ranged from 42.2 % (47.9 %) to 87.5 % (93.5 %) for the remainder of the study period. GM live tick counts for both products were significantly lower (*P ≤* 0.0011) than placebo on all days, and significantly fewer (*P ≤* 0.0415) live ticks were found on sarolaner-treated dogs compared to fluralaner-treated dogs from Day 44 onwards. There were no treatment-related adverse reactions during the study.

## Discussion

Sarolaner significantly reduced *R. sanguineus* tick counts within 8 h after the first treatment on Day 0 and demonstrated a consistently high efficacy within 24 h after 3 monthly treatments. In contrast, the speed of kill of fluralaner was significantly slower from Day 44 onwards. These results are consistent with a previous study in which the speed of kill of fluralaner against *R. sanguineus* and *Dermacentor reticulatus* also decreased in the third month after treatment [15]. In the present study, significantly more *R. sanguineus* ticks were found on the fluralaner-treated dogs from Day 44 onwards compared to dogs treated monthly with sarolaner. These results illustrate that the perceived benefit of a longer treatment interval and hence the need for less treatments, needs to be balanced with the potential risk of an unpredictable decline in efficacy at the end of the claimed treatment period. An important benefit of monthly sarolaner administration is that it provides a sustained efficacy against all relevant tick species in dogs [11] as well as maintaining a rapid speed of kill for the entire duration of the efficacy claim period against *R. sanguineus* and other tick species (*I. ricinus*, *I. scapularis, D. reticulatus* and *A. maculatum*) [12, 13]. As *R. sanguineus* is the vector of a range of tick-borne diseases worldwide, the rapid and consistent speed of kill provided by monthly treatments with sarolaner will be an important aid in the prevention of tick-borne disease transmission.

## Conclusions

This study confirmed the consistent acaricidal efficacy of sarolaner against *R. sanguineus* and demonstrated that ticks were killed rapidly during the entire treatment period. Monthly treatment with sarolaner consistently killed more ticks within 24 h than a single dose of fluralaner from 6 to 13 weeks after initial treatment.
